# "Brace technology" thematic series - the Gensingen brace™ in the treatment of scoliosis

**DOI:** 10.1186/1748-7161-5-22

**Published:** 2010-10-13

**Authors:** Hans-Rudolf Weiss

**Affiliations:** 1 1 Orthopedic Rehabilitation Services, D-55457 Gensingen, Alzeyerstr. 23, Germany

## Abstract

**Background:**

Bracing concepts in use today for the treatment of scoliosis include symmetric and asymmetric hard braces usually made of polyethylene (PE) and soft braces. A new asymmetric Chêneau style CAD/CAM derivate has been designed to overcome problems the author experienced with other Chêneau CAD/CAM systems over the recent years.

**Brace description:**

This CAD/CAM Chêneau derivate has been called Gensingen brace™, a brace available to address all possible curve patterns. Once the patients' trunk is scanned with the help of a whole trunk optical 3D-scan and the patients' data from the clinical measurements are recorded, a model of the brace can be created by (1) modifying the trunk model of the patient 'on screen' to achieve a very individual brace model using the CAD/CAM tools provided or by (2) choosing a brace model from our library and re-size it to the patients' properties 'on screen'.

**Results:**

End-result studies have been published on the Chêneau brace as early as 1985. Cohort studies on the Chêneau brace are available as is a prospective controlled study respecting the SRS criteria for bracing studies, demonstrating beneficial outcomes, when compared to the controls using a soft brace. Sufficient in-brace correction effects have been demonstrated to be achievable when the Chêneau principles of correction are used appropriately. As there is a positive correlation between in-brace correction and the final outcome, the Chêneau concept of bracing with sufficient in-brace corrections as published can be regarded as being efficient when applied well. Case reports with high in-brace corrections, as shown within this paper using the Gensingen brace™ promise beneficial outcomes when a good compliance can be achieved.

**Conclusions:**

The use of the Gensingen brace™ leads to sufficient in-brace corrections, when compared to the correction effects achieved with other braces, as described in literature.

According to the patients' reports, the Gensingen brace™ is comfortable to wear, when adjusted properly.

Further studies are necessary (1) in order to evaluate brace comfort and (2) effectiveness using the SRS inclusion criteria.

## Introduction

Bracing concepts in use today for the treatment of scoliosis include symmetric and asymmetric hard braces usually made of PE on the one hand and soft braces on the other. The latest developments in the field of bracing, aim at (1) improving specificity with respect to the individual curve pattern of the patient treated and (2) at a restoration of a proper sagittal realignment [1,2].

Although the effect of brace treatment has been questioned [3], there is evidence that brace treatment can stop curvature progression [4-9], reduce the frequency of surgery [10-12] and improve cosmetic appearance [13-15]. Poor cosmetic appearance for the patient may be the most important problem, which can be solved or at least reduced by the use of advanced bracing techniques including the best possible correction principles available to date [13].

**Figure 1:**
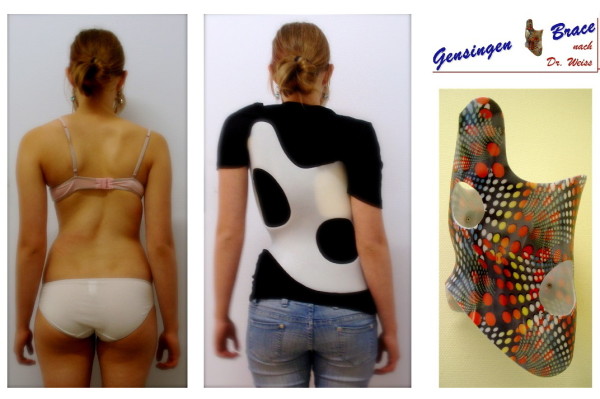
**Gensingen brace™ for the correction of a functional 4-curve scoliosis**.

The plaster cast method worldwide seems to be the most practiced technique for the construction of hard braces at the moment. CAD (Computer Aided Design) systems are available, which allow brace adjustments without plaster. Another new development is the ScoliOlogiC™ off the shelf system enabling the technician to construct a light brace for scoliosis correction from a variety of pattern specific shells to be connected to an anterior and a posterior upright [1]. This Chêneau light™ brace, constructed according to the Chêneau principles using the brace parts from the ScoliOlogiC™ off the shelf system, promises a reduced impediment of quality of life in the brace. A satisfactory in-brace correction exceeding 50% of the initial Cobb angle has been achieved with this brace [2], which was used as the basis for the development of the Gensingen brace™ (Fig. 1).

### History of the Gensingen brace™

**Figure 2:**
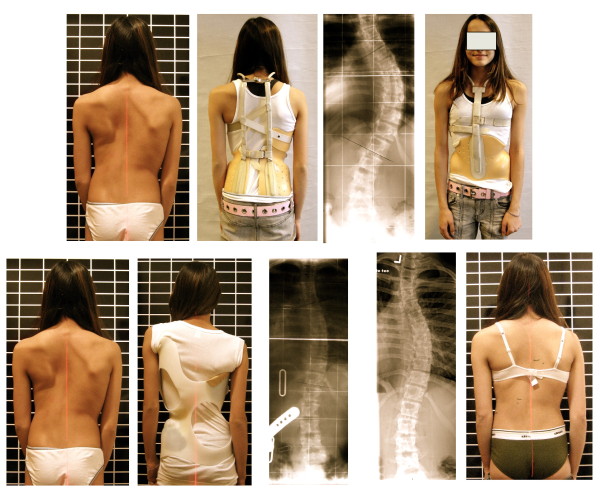
**Patient from North America with a thoracic curve of 56° initially treated with a Milwaukee brace (upper line of pictures)**. The curve corrected from 56 to 53°, only. The same curve has been corrected to 27° in a Chêneau brace with a good clinical and cosmetic intermediate result. Nevertheless, the patient from North America finally decided to have spinal surgery, although the Chêneau brace treatment seemed promising.

The Gensingen brace™ is a Chêneau derivative in principle. The Chêneau brace was developed before 1978 [16]. As the first developments were made in Münster, Germany, the brace was initially called CTM-brace (Chêneau-Toulouse-Münster). Jacques Chêneau, who used to live in Toulouse, spent a few years in Münster, where he braced patients at the orthopedic department of the university. In 1985 the first end-result study was published with in-brace correction effects of more than 40% of the initial value [7] and final results superior to the end-results of the Milwaukee study from the same centre [17] (Fig. 2). The initial Chêneau brace was upgraded in 1995 and from this year on, a new version each year was promoted by the inventor during the courses organized in Germany together with Dr. Weiss in Bad Sobernheim and Prof. Neff in Berlin. A working relationship between Dr. Chêneau, Dr. Weiss and Dr. Rigo began in Bad Sobernheim towards the end of the 90's, which resulted in a collaboration publishing a book presenting the 1999 standard of the Chêneau brace [18].

At the beginning of the new century Dr. Chêneau was working on the first CAD/CAM system supported by a company called IPOS.

Other CAD/CAM systems developed in Germany applying the Chêneau principles, such as the Regnier™ system and the RSC™-brace.

In the summer of 2006 the fabrication of the ScoliOlogiC^® ^off the shelf bracing system for the adjustment of Chêneau light™ braces began.

The Chêneau light™ brace is available for right thoracic and left lumbar curvatures, only. For thoracolumbar curvatures no Chêneau light™ shells are available. Another limitation for the application of the Chêneau light™ brace is the limited number of shell sizes not allowing to brace patients with small trunks as well as in children aged ten or under.

Therefore, in our department it has been necessary to use plaster based bracing or a CAD/CAM system in addition to the Chêneau light™ brace.

At first, we considered using a CAD/CAM system that was already available. However, the Regnier™ system still today does not address the sagittal profile accordingly and the RSC™ braces still have a high percentage of rotation instability [19]. The latter may lead to a loss of the correction initially achieved after a few weeks of brace wearing [19].

**Figure 3:**
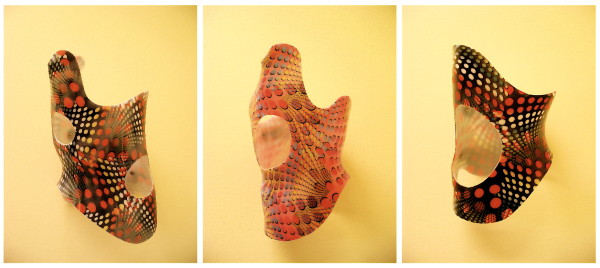
**Three examples of Gensingen braces™**. On the left there is a brace to address a double curve (3CL, 4C), in the middle, a brace to address single thoracic curves (3C, 3CH) and on the right, a brace for the treatment of a thoracolumbar curve pattern.

**Figure 4:**
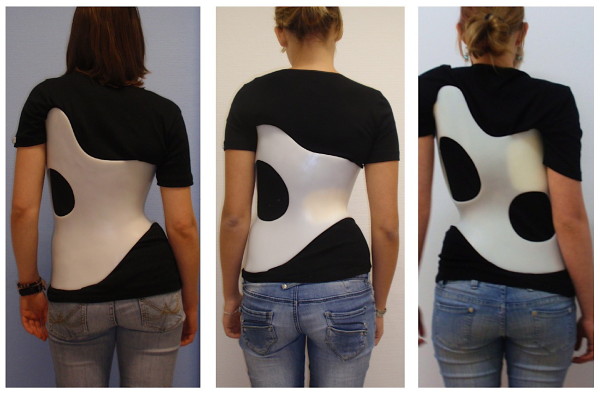
**Different Gensingen braces™ on the patient's body**. On the left for a 3C scoliosis: The decompensation to the right has been overcorrected with the trunk being hyper compensated to the left in the Gensingen brace™ 3C. The middle picture shows a patient with right lumbar scoliosis in a Gensingen brace™ 4CL. The pelvis is hyper compensated from left to the right and the thoracic counter curve seems well balanced in the brace. On the right, a patient in a Gensingen brace™ 4C is documented. Pelvis is hyper compensated from right to left and the thoracic area is hyper compensated in the same direction.

Therefore, with the help of Orthomed Scoliocare, Orthopedic Technical Services in Gensingen a new CAD/CAM system was developed in Spring 2009 with the aim to overcome the shortcomings of the CAD/CAM systems already available in Germany and to enable brace adjustments for patients of all possible curve patterns (Fig. 3 and 4) and trunk sizes.

**Figure 5:**
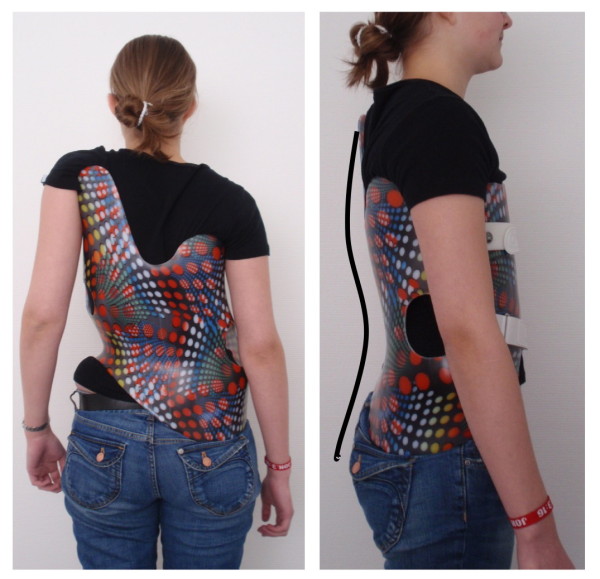
**Sagittal alignment of a Gensingen brace™ 4C**. On the right the implementation of a physiologic sagittal profile is visible with a pronounced lumbar lordosis and a slight thoracic kyphosis.

A slight lumbar lordosis has been introduced into the braces included into our library (Fig. 5), which can be augmented by using foam pads and the "Stop Point" against rotational forces (Point 37 according to Chêneau) has been set laterally in order to have a better relation to the anterior superior iliac spine on the rib-hump side.

Although, dependent on the pelvic geometry of the individual patient, we are still experiencing some rotation instabilities in the new design, we have been able to reduce this problem drastically.

### Theoretical principles

Many 3-point pressure systems are applied on the frontal, coronal and sagittal plane as in all other Chêneau derivates. Opposite every pressure area an expansion void is implemented. This enables the desired corrective movement and - when adjusted properly - avoids compression effects leading to pressure sores. As a matter of fact, in today's Chêneau braces pressure sores have become a very rare complication.

The brace action is demonstrated on morphing videos, which can be seen on the links provided: http://www.youtube.com/watch?v=sd9mbyzXflQ and http://www.youtube.com/watch?v=9uNZmY6jQas.

Pattern specific bracing is desirable to allow the correction of the individual curve patterns appropriately, as theoretically there might be an unlimited number of curve patterns with different geometrical entities. Therefore, a classification is necessary to come as close as possible to the individual pattern of the patient in order to address the biomechanical properties of the individual curve pattern of the patient treated.

After the first curve patterns were identified by Ponseti and Friedmann [20], and Moe and Kettleson [21] for surgical means, in the late 70's a simple functional classification for approaching different curve patterns with the help of physiotherapy was established by Lehnert-Schroth [22,23]. This classification simply distinguished between so called (functional) 3- and 4-curve patterns.

Chêneau also used this simple classification for the construction of his braces.

The King classification [24] distinguished between 5 different (thoracic) curve patterns and was established in the 80's to help the surgeons to approach the curves properly during operations.

The Lenke classification [25], which is rather complex was developed by surgeons, because the use of the King Classification had lead to imbalanced post surgical results and seemed to lack reliability.

Rigo [26] implemented a new classification for brace treatment with 15 different curve patterns, derived from the Lenke classification [25]. All those curve patterns - according to his opinion - demand individual principles of correction in 3D, however, 5 key patterns have been identified which we have started working with in everyday practice [27].

This year Rigo came up with a brand new classification [28], which, in our opinion is not simple enough to be used by technicians and CPOs generally.

**Figure 6:**
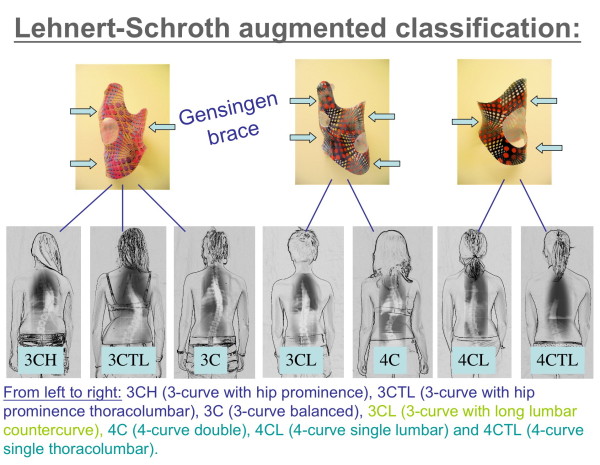
**The augmented classification according to Lehnert-Schroth**. Curvatures decompensated to the thoracic convex side have to be regarded as functional 3-curve type curvatures, when balanced or decompensated to the thoracic concave side (lumbar curves as big as thoracic ones or even bigger) per definition they are functional 4-curve types. As can be seen, the 3-curve lumbar is a 3-curve pattern, but treated like 4-curve with reduced correction in the lumbar curve.

Therefore, at our center we are now returning to the simple functional classification by Lehnert-Schroth [22,23]. The principal subdivision of functional 3 and functional 4 curves still seems to work for physiotherapy and can easily be augmented to the needs of the technician or CPO. This augmented Lehnert-Schroth classification [29] can be seen on Fig. 6 as well as the braces used to address the individual curve patterns.

### Brace description

The advantage of the Gensingen brace™ is that the brace is available in a short time (3 days with overnight milling service, or even shorter), is easily adjustable and is very comfortable to wear, because many compression effects - in frontal and sagittal plane as well - apparent within other specific, non symmetric CAD/CAM systems - have been ruled out in this system of bracing. As a matter of fact many other CAD/CAM Chêneau derivates lack a balanced distribution of pressure areas.

**Figure 7:**
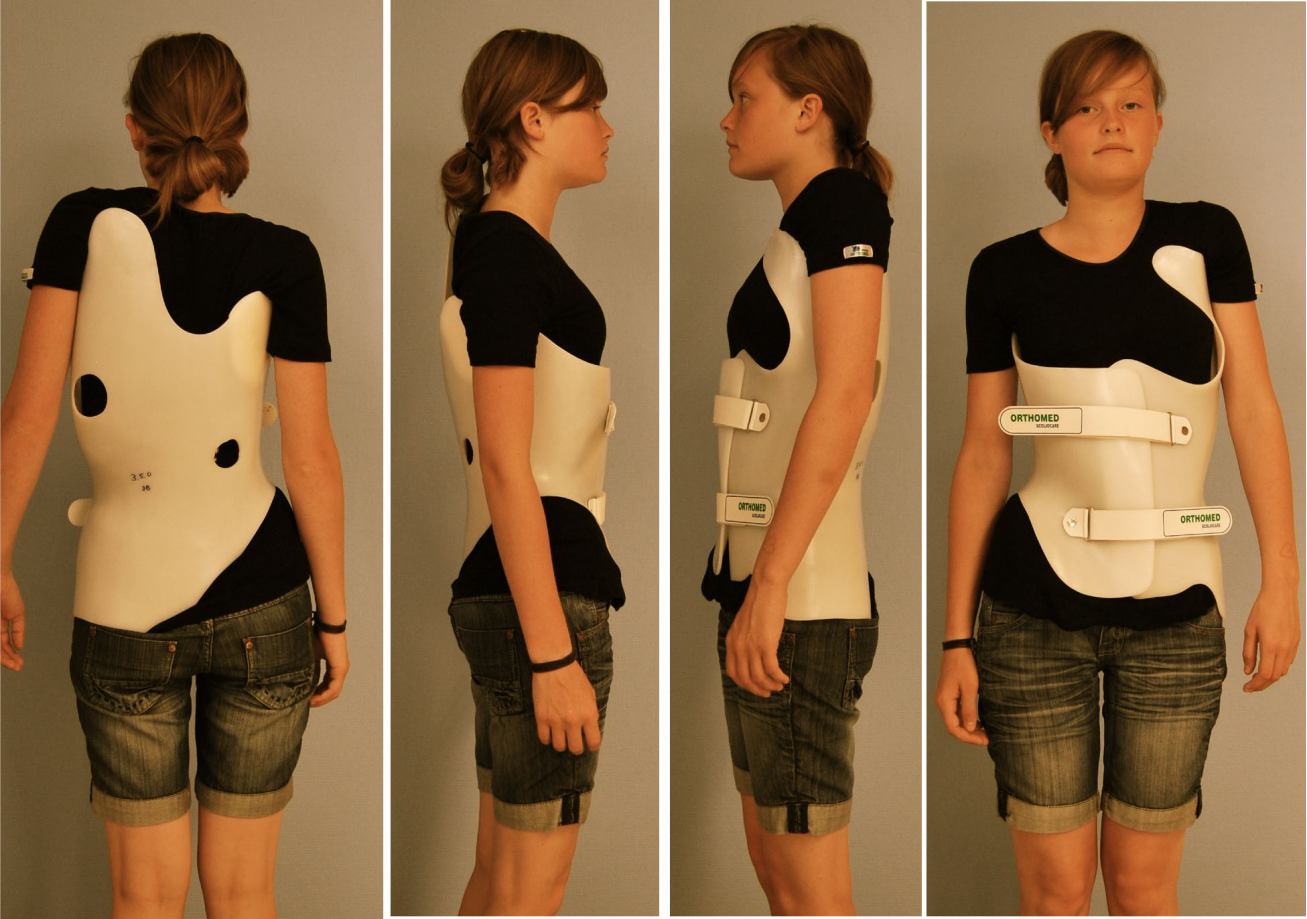
**A brace to address functional 3-curve patterns for right thoracic curves from all four sides**. The static overcorrection to the concave side is already visible in this "try-on" brace not yet cut and finalized. A final 3-curve brace can be seen on Fig. 4. on the left. The final brace is much smaller than the 'try on' brace.

**Figure 8:**
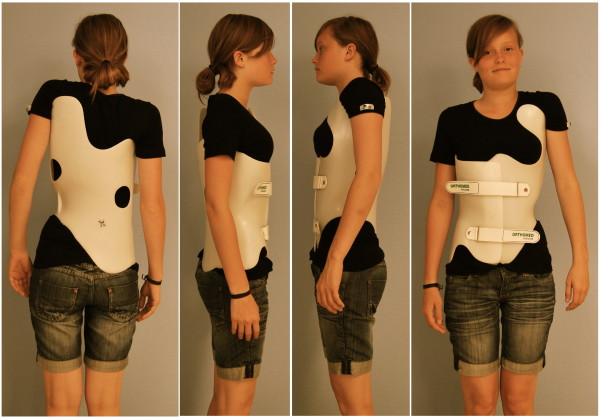
**A brace to address functional 4-curve patterns for right thoracic and left lumbar curves from all four sides**. The static recompensation of the trunk segment is already visible in this "try-on" brace not yet cut and finalized. A functional 4-curve pattern brace in its final form can be seen on Fig. 4 on the right. The final brace is much smaller than the 'try on' brace.

**Figure 9:**
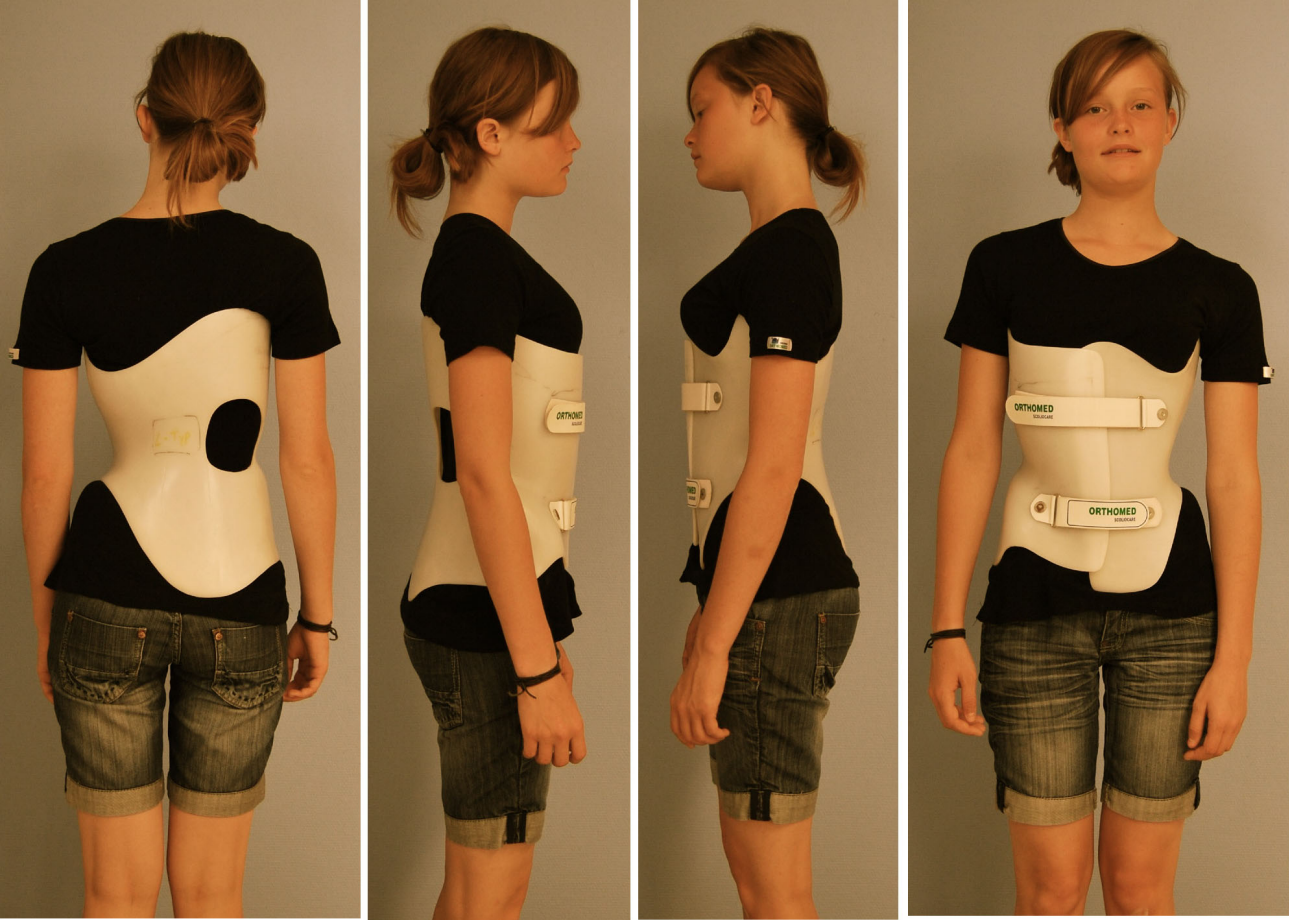
**A short brace to address the thoracolumbar curve pattern 4CTL**. The static overcorrection to the thoracolumbar concave side is already visible in this "try-on" brace not yet cut and finalized with pelvic prominence mirrored. A final 4C-curve short brace can be seen on Fig. 4. middle. The final brace is much smaller than the 'try on' brace.

Braces to address functional 3-curve patterns (Fig. 7) and braces to address functional 4-curve patterns (Fig. 8) are available, as are braces for thoracolumbar curve patterns (Fig. 9). There are more than 35 different braces in our library including models for athletic trunks as well as models for small children. No extra models are used for double thoracic curvatures as these easily can be addressed by fine adjustment of the models from the library.

The brace is usually adjusted to the patients' body with the help of the pattern specific blueprints (Additional file 1.) as a raw "try on" brace first as it has been cut from the foam positive (Fig 7, Fig 8, Fig 9). The construction of the Gensingen brace™ from the milling of the foam positive to the fine adjustment to the patient's body can be seen on the first part of the video on the link: http://www.youtube.com/watch?v=0P9eKW0Fpis.

### Practical issues

#### How to prescribe the brace

The Gensingen brace™ at our centre is not prescribed per se. The prescription does not contain the specific brace name, but a Chêneau brace is prescribed and the curve pattern of the individual patient is also submitted. The Cobb angles of all curvatures should also be visible on the prescription.

Additionally, a construction plan (e.g. in Additional file 2.) for the brace prescribed is attached to the prescription and should a brace have to be renewed, a further sheet giving justification (Additional file 3.) as to why a new brace has to be prescribed should be included.

**Figure 10:**
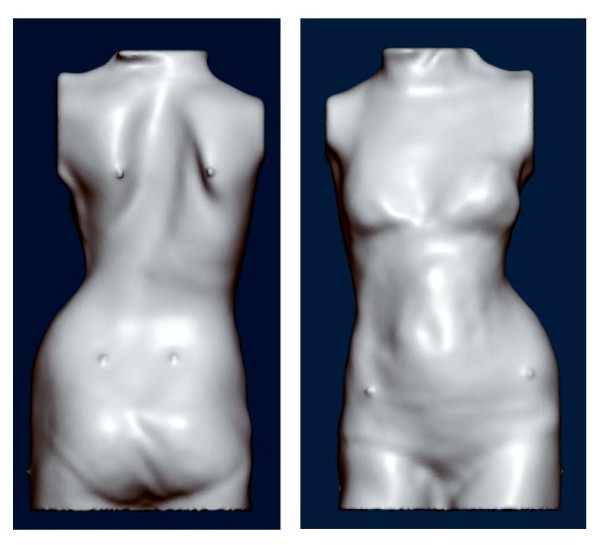
**Whole trunk scan of a patient with right thoracic scoliosis**. The decompensation is clearly visible. To paste some markers on the trunk before the scan is taken makes it easier to identify special landmarks of the trunk while modifying the trunk model in the computer with the help of the CAD/CAM tools provided.

With this Chêneau prescription, the patient can go to any workshop near home to get his or her brace done using plaster casting, CAD/CAM system braces or Chêneau light braces. If the patient makes the decision to go to the workshop in Gensingen, the CPOs show diverse possible Chêneau derivates so as to enable the patient to decide on the brace type her- or himself. When the Gensingen brace™ has been chosen the CPO firstly performs a trunk scan (Fig. 10) and then starts to take the measurements necessary for proper brace adjustment as has been demonstrated on the linked video: http://www.youtube.com/watch?v=0P9eKW0Fpis.

#### How to build the brace

Once the patients' trunk is scanned with the help of a whole trunk optical 3D-scan and the patients' data from the clinical measurements are recorded, a model of the brace can be created by (1) modifying the trunk model of the patient 'on screen' to achieve a very individual brace model using the CAD/CAM tools provided or by (2) choosing a brace model from our library and re-size it to the patients' properties 'on screen'. In rare curve patterns the individual correction of the trunk scan is used, in curve patterns classified easily, the second procedure is usually chosen. In this case the patient has the advantage to receive a fully developed brace, where all known disadvantages of other bracing systems have already been ruled out as far as possible [19]. Individual modelling is not as safe with respect to in-brace correction and may be more uncomfortable at first. A visual impression on how the model is milled, vacuumed and how the brace is finally adjusted can be found on the linked video: http://www.youtube.com/watch?v=0P9eKW0Fpis.

#### How to check the brace

The brace is checked in a standardized way. First of all a verification of the pattern specificity is necessary, after that the pad (pressure area) attachment is checked clinically for the right height in relation to the neighboring pressure areas and voids. The voids are then controlled for the impact the construction clinically might have on the patient with respect to pain and other discomfort. This is done by the CPO first and is documented on the "Checklist" (Additional file 4.)

After that, the CPO presents the patient and the checklist to the physician, who has another checklist (available in German only, Additional file 5.) for the final clinical check-up.

**Figure 11:**
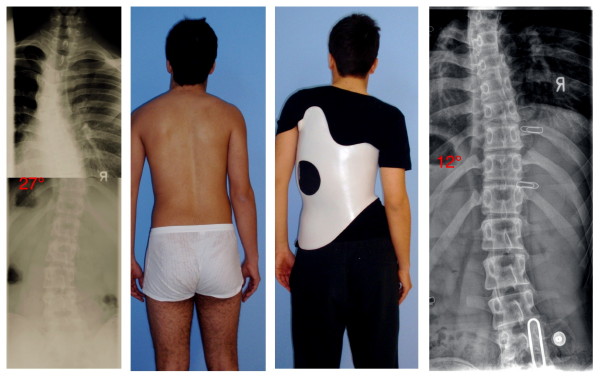
**14-year old boy in his second Gensingen brace™ for the treatment of a double thoracic curvature**. The initial angle of curvature at the start of treatment, when the boy was 12 years old, was 35° main thoracic with a significant high thoracic counter curve exceeding 30° as well. Now in his second Gensingen brace™, version 2010 for the treatment of a double thoracic curvature we have achieved a correction from 27° (intermediate result) to 12° in the main thoracic curvature, which is excellent considering the fact, that double thoracic curves cannot be corrected as easy as single thoracic curves. The foam pad location is marked on the in-brace x-ray with wire as is the longitudinal width of the thoracic pad.

**Figure 12:**
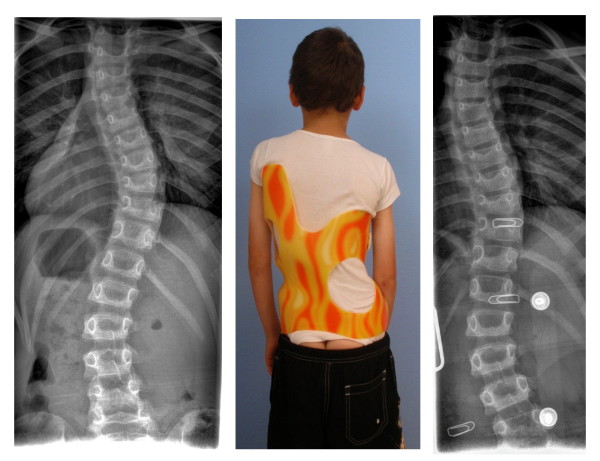
**10-year old boy with EOS (early onset scoliosis) in his first Gensingen brace™**. Clinically in this boy the flatback is clearly visible. The trunk is rather balanced because of the re-compensation of the 39° thoracic curve by a structural high thoracic counter curve. The curve is very stiff and has a bad prognosis, which can be estimated from the x-ray: In the apical area of the thoracic curve a significant wedging of the vertebra is visible. Nevertheless the curve has been corrected to 18° in the brace, which is sufficient considering the fact that double thoracic and EOS curves cannot be corrected as easy as single thoracic curves in patients with AIS. The foam pad location is marked on the in-brace x-ray with wire as is the longitudinal width of the thoracic pad.

**Figure 13:**
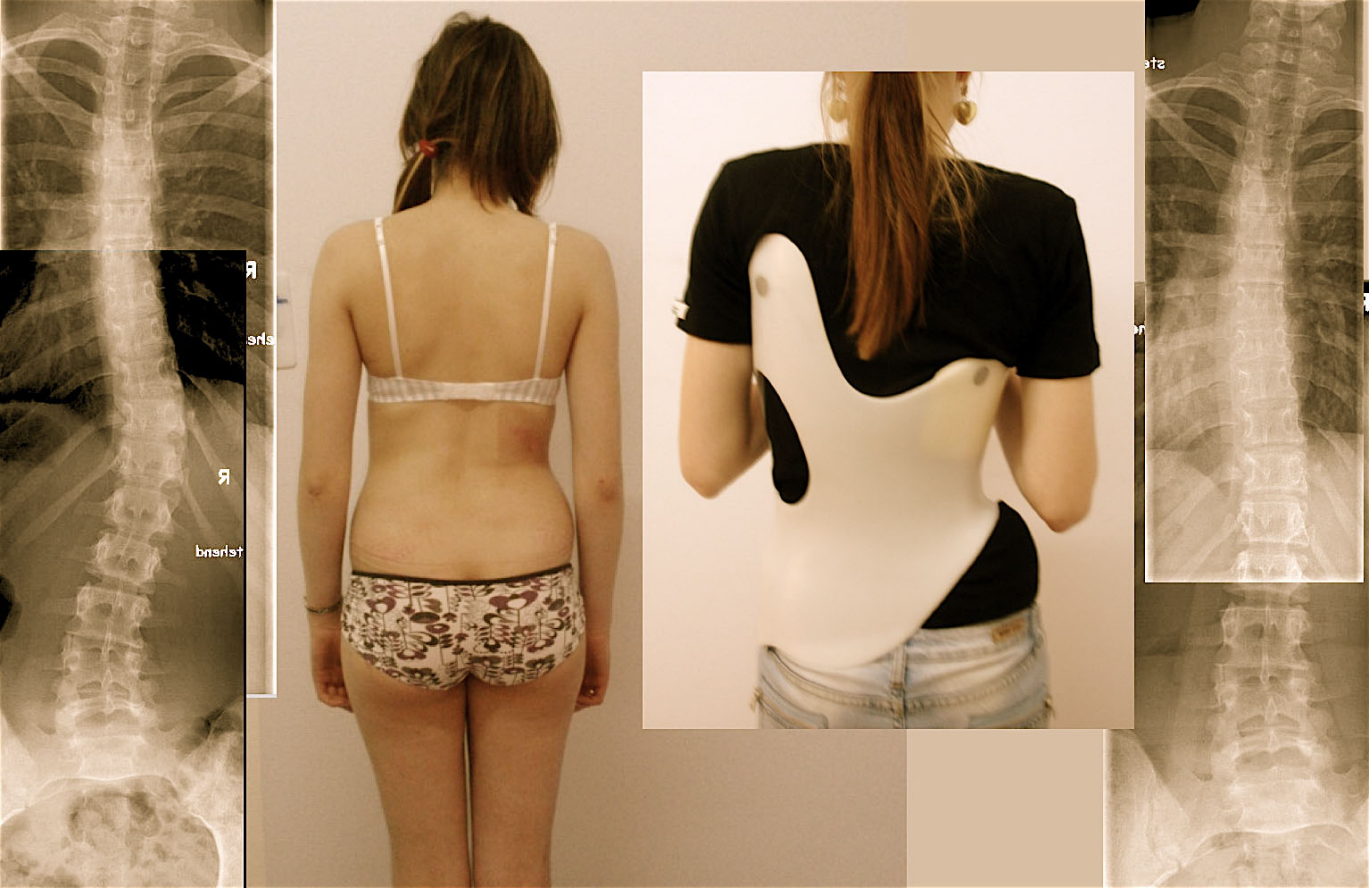
**Girl with AIS treated with a 3CTL brace**. The clinical picture on the left was taken after 6 weeks of brace wearing. The initial decompensation of the trunk has already disappeared. On the right in the brace the hyper compensation is demonstrated in order to allow a shift of the apical vertebra towards the midline. The x-ray result is sufficient with a >75% correction of the initial Cobb angle (left) in the Gensingen brace™ 3CTL.

After the necessary improvements have been made, the patient is scheduled for the next appointment in 6 weeks in order to have a clinical check-up and the in-brace x-ray completed using pad markers (Fig. 11, 12, 13).

#### Protocols

The criteria for bracing are taken one to one from the SOSORT indication guidelines [30].

#### Everyday usage

the number of hours per day that the patient should wear the brace in principle is taken one to one from the SOSORT indication guidelines [30].

#### Exercises

There are no exercises done in the brace because we aim at maximum possible correction giving no room for further corrections with the help of exercises. However, for the exercises without the brace on, the augmented Lehnert-Schroth classification is also applied (http://www.youtube.com/watch?v=eHsCsL7IEaU).

## Results & case reports

At least in Germany the Chêneau brace has been widely reviewed. As early as 1985 the first end-result study was published [7]. The average in-brace correction reported on within this study was 40%. Landauer [6] presented a case series of patients treated with the Chêneau brace with comparable in-brace corrections and comparable end-results.

A prospective controlled study comparing the Chêneau brace with the SpineCor has clearly shown the superiority of the Chêneau brace in a sample of patients at actual risk for being progressive, fulfilling the SRS criteria for studies on bracing [9]. After growth only 8% from the SpineCor sample and 80% of the Chêneau group were not progressive. The Cobb angle at the start of treatment however, was 21° for the SpineCor sample and 33° for the Chêneau brace sample of patients.

According to Landauer and collaborators, [6] two factors are influencing the outcome of brace treatment, both of them being equally important: In-brace correction (1) clearly correlates with the final result. The better the in-brace correction, the better the end-result. Compliance (2) is the other important factor. The best possible in-brace correction will not change the prognosis of the patient when the brace is not worn as prescribed.

In-brace corrections exceeding 50% have been reported in literature in a sample of patients treated with the Chêneau light™ brace having an average Cobb angle of 36° [2]. The Gensingen brace™ is adjusted according to the same principles of correction as the Chêneau light™ brace [31], therefore we expect similar results in both brace types.

As the majority of patients treated in our department choose the Chêneau light™ brace, the Gensingen brace™ is used in curvature patterns a Chêneau light™ brace is not available for, or for curvatures exceeding 50°. Therefore, a direct comparison of the results achieved with both brace types will not be possible in the near future.

**Figure 14:**
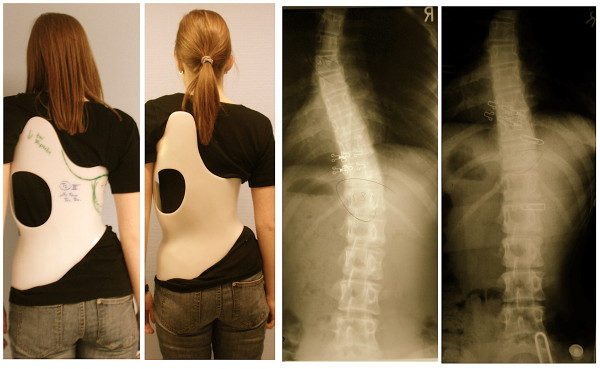
**Girl with double thoracic AIS treated with a 3C brace**. On the left the old 2009 model was used initially, on the second picture from left the final 2010 version of this Gensingen brace™ 3C was finally applied and the recompensation of the curve with a sufficient in-brace correction effect can be seen on the right.

**Figure 15:**
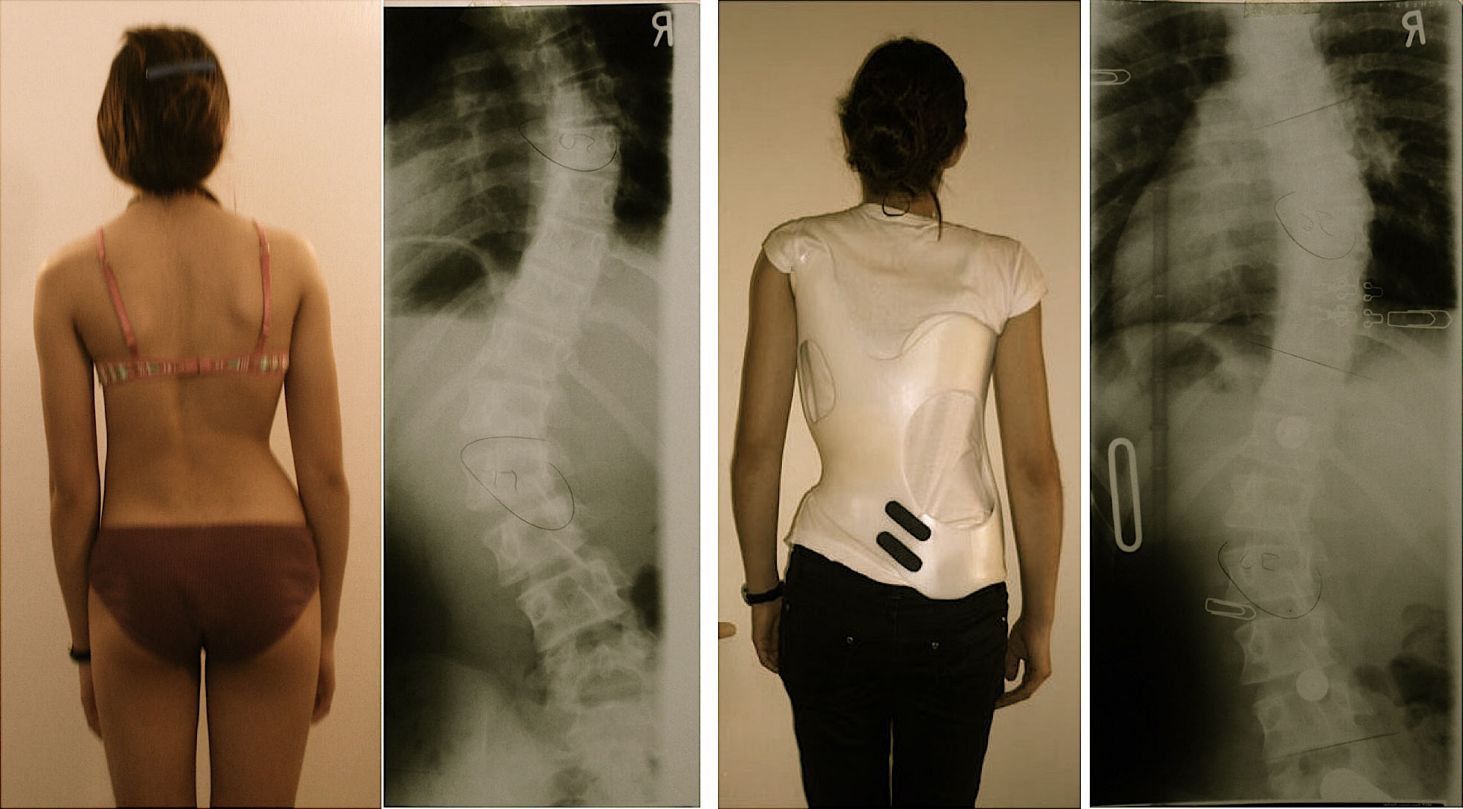
**Tall 12-year old girl from Denmark with double major curvature treated with a Gensingen brace™ 4C**. The curve of initially 50° thoracic and 55° lumbar has been corrected to 36° thoracic and 30° lumbar although this specific curve pattern with high lumbar and middle to low thoracic apex usually corrects least.

**Figure 16:**
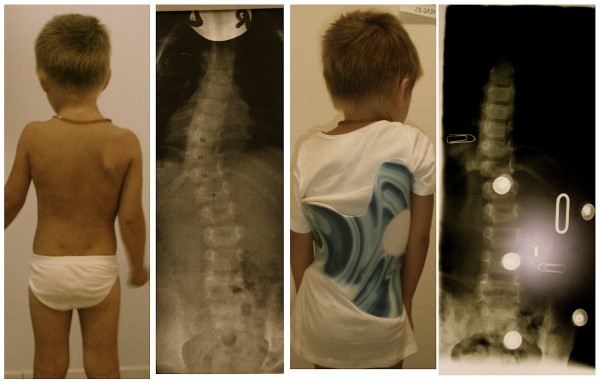
**Boy with an infantile curvature from Italy treated with a Gensingen brace™ 3C**. Treatment began with a Chêneau brace at the age of 4 years when the boy had 40°. Intermediate result: 28° corrected to 6° in the a Gensingen brace™ 3C

**Figure 17:**
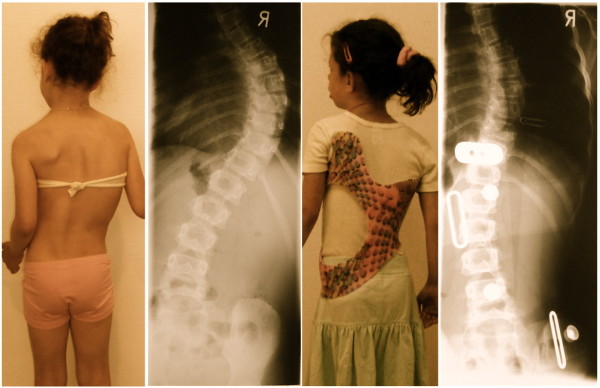
**12 year old girl with neuromuscular scoliosis treated with a Gensingen brace™ 3C**. The curvature exceeding 60° at the start of the treatment has been reduced by approximately 50% in the Gensingen brace™ 3C.

Nevertheless, we have documented case reports showing sufficient in-brace corrections in certain curve patterns and in bigger curves as well (Fig. 11, 12, 13, 14, 15, 16, 17).

According to the patients' reports the Gensingen brace™ is comfortable to wear, when adjusted properly.

## Discussion

Several bracing concepts are used today for the treatment of scoliosis and the in-brace corrections accepted as sufficient vary widely. The plaster cast method worldwide seems to be the most practiced technique at the moment. CAD systems are available which allow brace adjustments without plaster. The latest development however, the ScoliOlogiC^® ^off the shelf system is not available for all patterns of curvature and for all possible trunk sizes.

**Figure 18:**
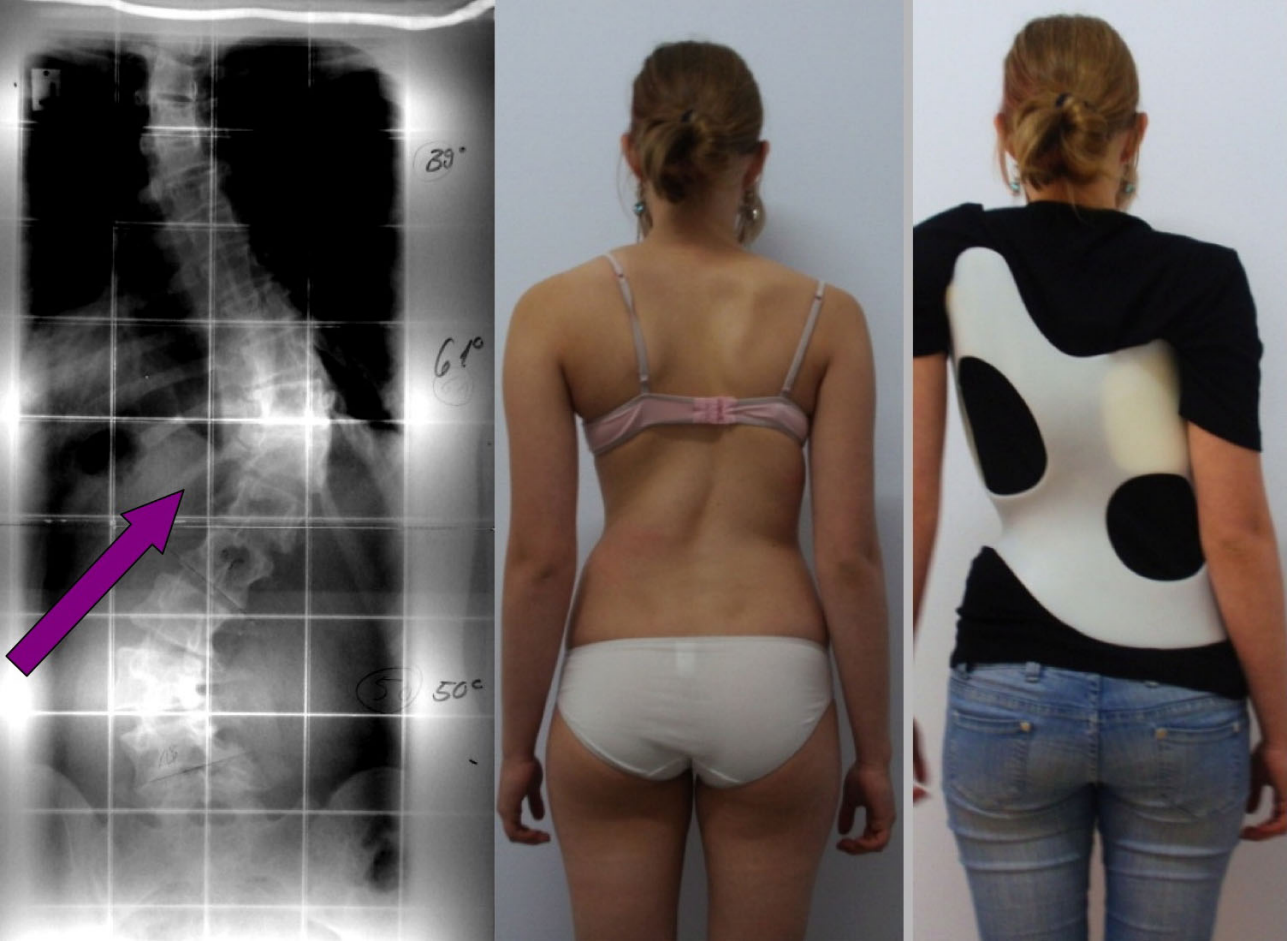
**14 year old girl with a thoracic curve exceeding 60° and with a short lumbar counter curve of 50° treated with a Gensingen brace™ 4C**. The decision was made to counter tilt the pelvis due to the size of the lumbar counter curvature. Although the clinical picture in the brace looks good we were not able to re-compensate the thoracic curve sufficiently because of a compression effect caused by the lower ribs included into the lumbar pressure area (arrow).

**Figure 19:**
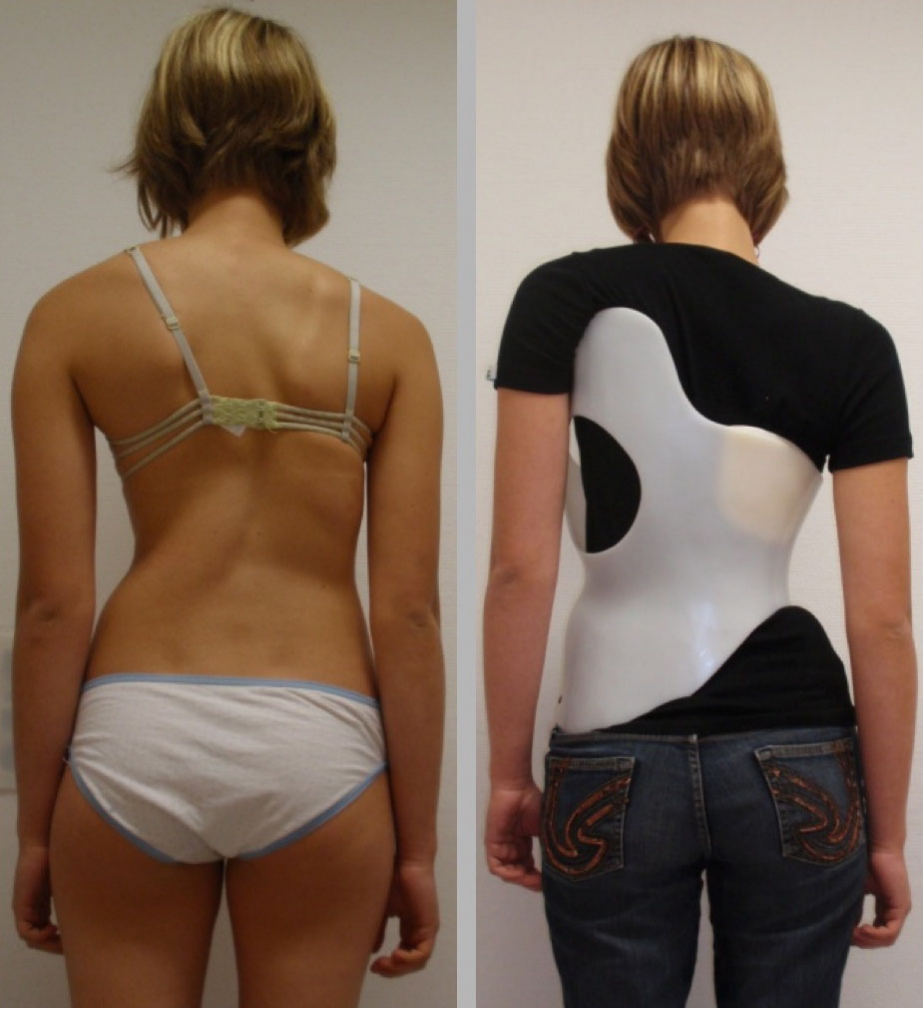
**Same patient as on Fig. 18 with changed brace pattern**. After 6 months of treatment with a Gensingen brace™ 4C the lumbar curve was reduced, however the thoracic curve had increased by 5°. This is why we decided to change the brace to a Gensingen brace™ 3C in order to get rid of the rib shift on the concave side against the thoracic re-compensation. This is to show that sometimes it is not clear from the very start which brace may be the best for the patient. Today we address the primary curve at first and risk an increase of the counter curve when a patient is imbalanced. This allows having the best possible cosmetic result at the very end; however in this patient, there was no more change of cosmetics. The patient, nevertheless, is satisfied cosmetically and refuses surgery.

Therefore, with the help of Orthomed Scolicare, Orthopedic Technical Services in Gensingen a new CAD/CAM system was developed in Spring 2009 with the aim to overcome the shortcomings of the CAD/CAM systems already available in Germany and to enable brace adjustments for patients of all possible curve patterns (Fig. 18 and 19) and trunk sizes.

In the normal range of brace indications a correction effect of at least 20% seems necessary to prevent progression [32], while a correction effect of an average 30% promises some final corrections [33]. A correction effect of 40% and more in a growing adolescent may lead to a final correction of an average 7° Cobb [6].

Wong et al. [34] reported correction effects of an average of 40% in patients with an average Cobb degree of 30, 6° (21° - 43°). However, in this collective, no patients with double curve patterns were included, which generally corrected worse than single curves in our preliminary study using the Chêneau light™ brace [35].

Bullmann et al. [36] reported average correction effects of 43% in the custom Chêneau brace constructed via plaster cast in patients with a Cobb angle of 31° (25° - 40°). The final rate of success in this study however, was only 58%, which has to be regarded as rather disappointing, when compared to the success rate of 80% we reported on in another prospective study [9] with an average correction effect of less than 40% in custom Chêneau braces constructed via plaster cast (prospective controlled study) and compared to the success rate of about 80% as reported in another independent prospective study [6].

No end result studies on the Gensingen brace™ are available at this stage, as this is a new application, but as it uses the Chêneau principles of correction comparable outcomes can be assumed as in the Chêneau braces investigated previously [6,7,9-11].

**Figure 20:**
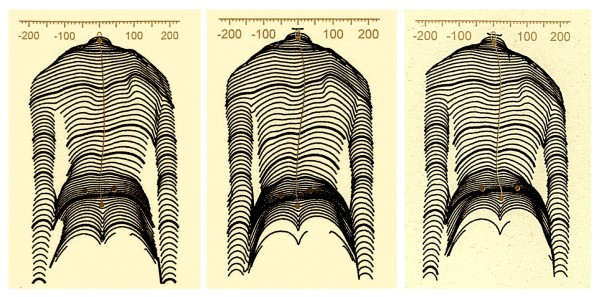
**Clinical course of a patient treated with the Gensingen brace™**. This 13 year old girl with a curvature exceeding 50° has been compliant after having received her first Gensingen brace™ in March at the first Scoliosis Short-Term Rehabilitation (SSTR) in Gensingen. She has been compliant and clearly shows an improvement of trunk asymmetry. This is clearly visible when comparing the left waist area after three months (middle) and after six months (right) of treatment time. Additionally she is exercising regularly.

As can be seen on Fig. 20 not only the Cobb angle, but also the clinical appearance of the patient with respect to trunk symmetry can be corrected by use of the Gensingen brace™ even in curvatures exceeding 50°.

## Conclusions

The use of the Gensingen brace™ leads to sufficient in-brace corrections, when compared to the correction effects achieved with other braces as described in literature. This has been demonstrated in the cases shown within this paper.

According to the patients' reports the Gensingen brace™ is comfortable to wear, when adjusted properly.

Further studies are necessary (1) to evaluate brace comfort and (2) effectiveness using the SRS inclusion criteria.

## Competing interests

The author is applying for a patent relating to the content of this paper (Chêneau light™ brace) and is advisor of Koob-Scolitech, Abtweiler, Germany (http://www.koob-scolitech.com).

## Authors' contributions

HRW: Manuscript writing, literature review, analysis and interpretation of data, preparation of the manuscript, acquisition of pictures and additional materials,

## Supplementary Material

Additional file 1 **PDF file containing the basic pattern specific blueprints according to the augmented Lehnert-Schroth classification**. Click here for file

Additional file 2 **Example of a construction plan as used in Germany**. These construction plans are included with German description and serve only for documentation purposes within this article. Click here for file

Additional file 3 **Short appraisal for justification for the new brace, sheet as used in Germany**. These appraisals plans are in German language and serve only for documentation purposes within this article. Click here for file

Additional file 4 **CPO's checklist as used in Germany**. The checklist is in German language and serves only for documentation purposes within this article. Click here for file

Additional file 5 **Physicians Checklist as used in German**. The checklist is in German language and serves only for documentation purposes within this article. Click here for file
