# Supplementary material for: "Brace technology" thematic series - the Gensingen brace™ in the treatment of scoliosis
Source: Scoliosis. 2010 Oct 13;5:22. doi: 10.1186/1748-7161-5-22 (PMC2967515; doi:10.1186/1748-7161-5-22)
Supplement: Additional file 1 — PDF file containing the basic pattern specific blueprints according to the augmented Lehnert-Schroth classification. [file 1748-7161-5-22-S1.PDF]

Blueprints for the pattern specific brace treatment of patients with scoliosis taking into consideration the correction principles of Chêneau and the patterns of the augmented classification of Lehnert-Schroth

Hans-Rudolf Weiss, MD

A detailed description of the key patterns can be found at:

*Hans-Rudolf Weiss: Befundgerechte Physiotherapie bei Skoliose.
3rd. Edition, Pflaum, Munich 2010*

© Hans-Rudolf Weiss, MD, 2010

3-curve with hip prominence (3CH)

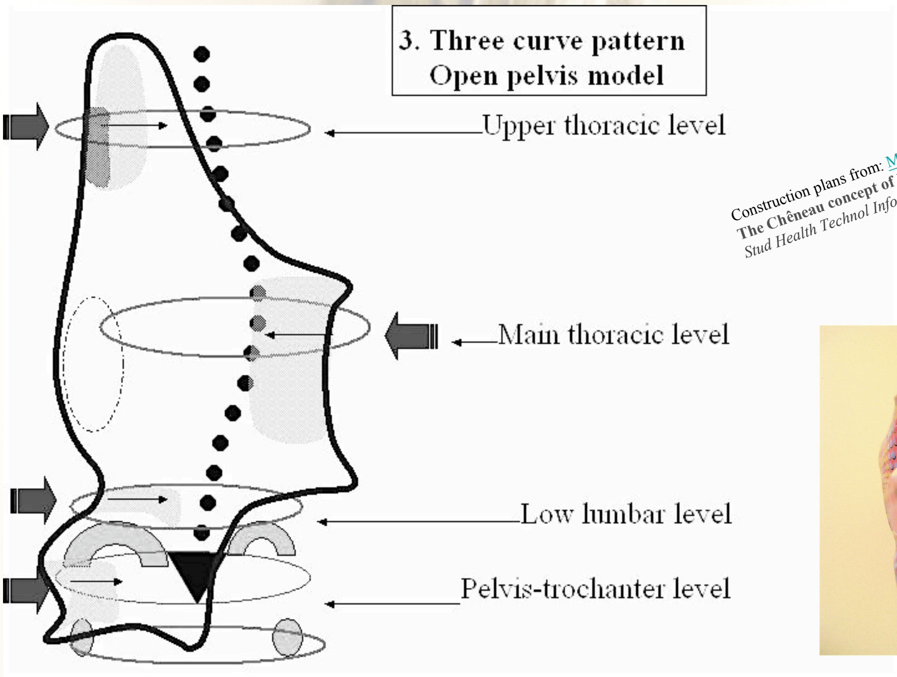

Construction plans from: Manuel Rigo, Hans-Rudolf Weiss (2008)
The Chêneau concept of bracing--biomechanical aspects.
Stud Health Technol Inform 135: 303-319

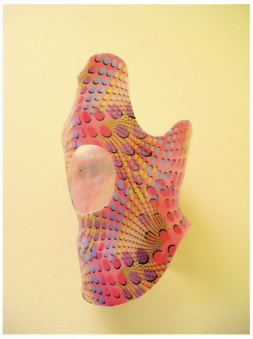

Example of treatment

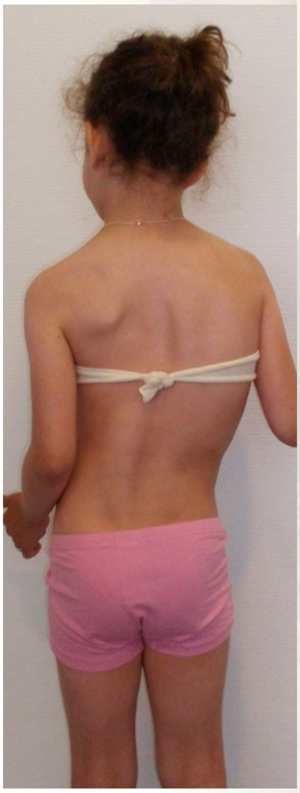

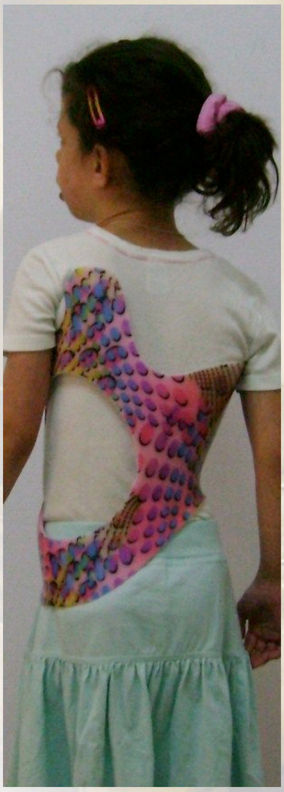

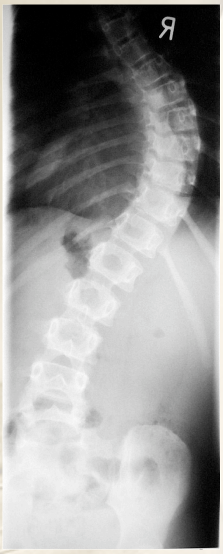

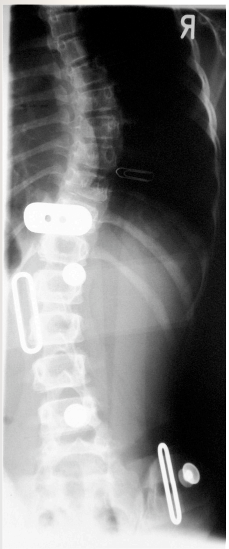

Girl with a 3CH pattern due to neuromuscular condition. Satisfying in-brace correction in a curve initially exceeding 60°.

© Hans-Rudolf Weiss, MD, 2010

3-curve balanced (3C)

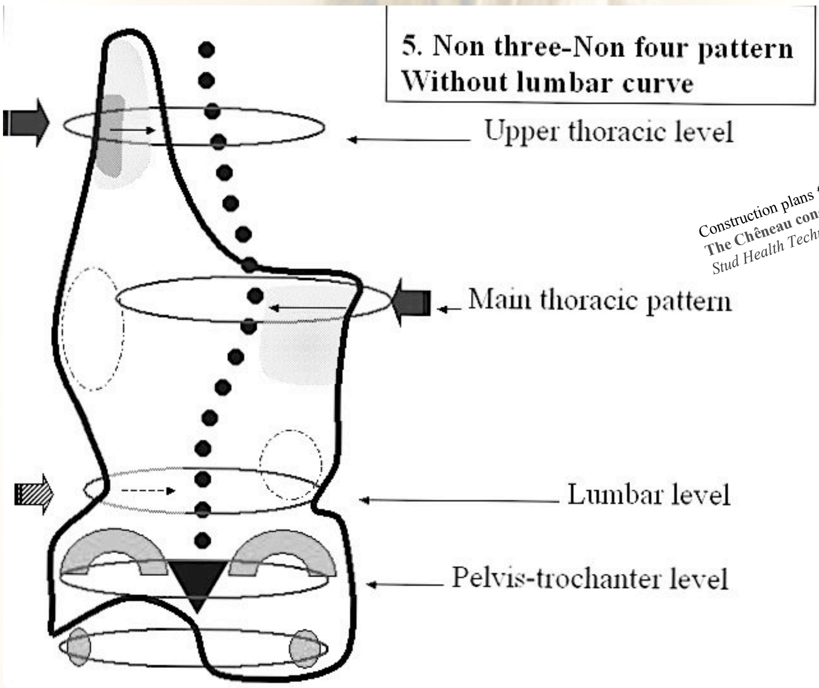

Construction plans from: Manuel Rigo, Hans-Rudolf Weiss (2008)
The Chêneau concept of bracing--biomechanical aspects.
Stud Health Technol Inform 135: 303-319

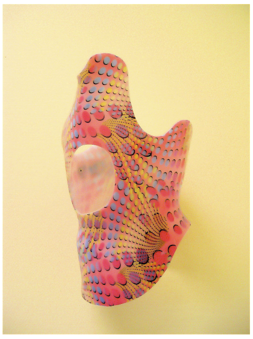

Example of treatment

Trochanteric pressure area may be omitted in modern well balanced braces!

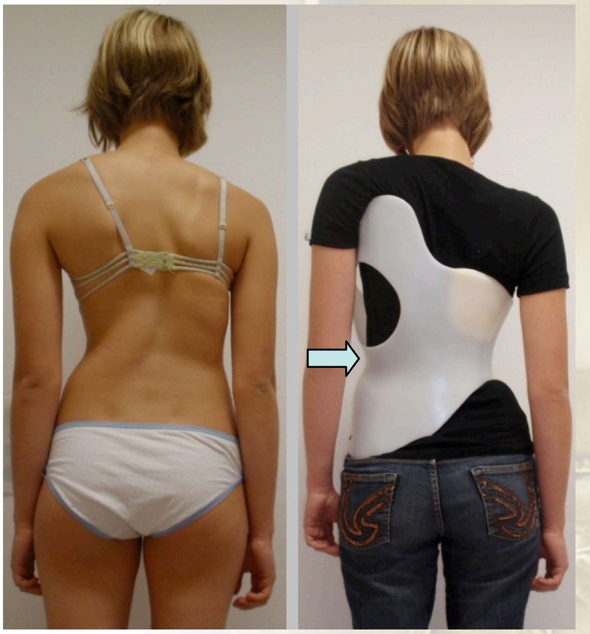

Curve balanced because of the high thoracic counter curve. To avoid a compression in the lower thoracic area the pad (arrow) has been omitted, but can be implemented, when the lumbar counter curve is longer.

© Hans-Rudolf Weiss, MD, 2010

3-curve with long lumbar curve (3CL)

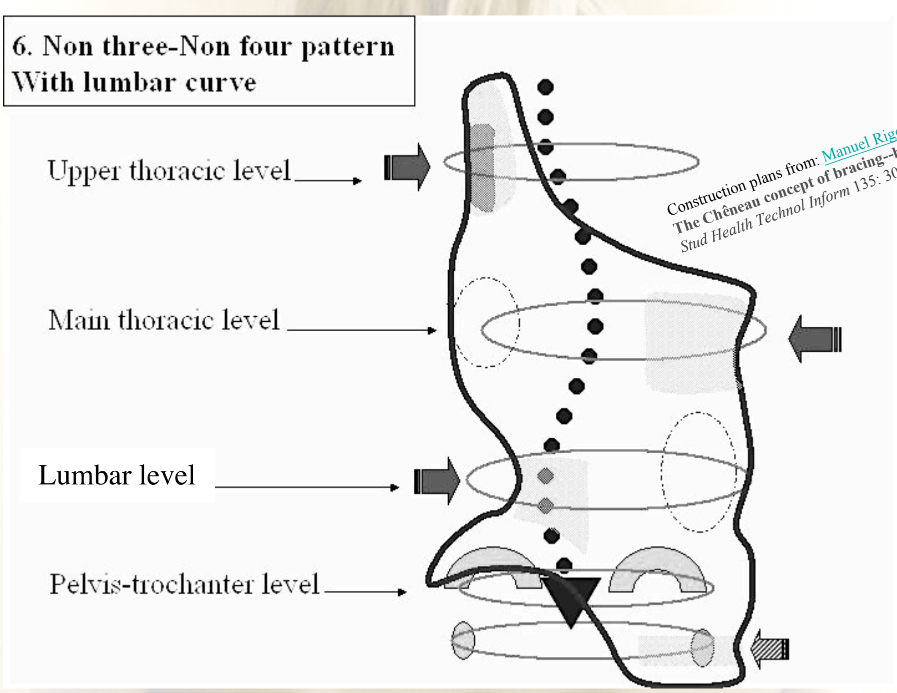

Construction plans from: Manuel Rigo, Hans-Rudolf Weiss (2008)
The Chêneau concept of bracing--biomechanical aspects.
Stud Health Technol Inform 135: 303-319

Example of treatment

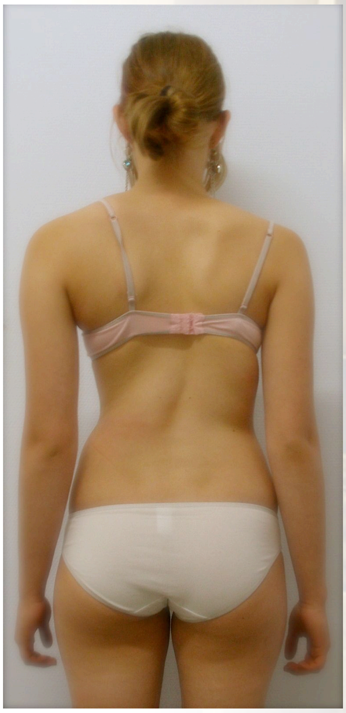

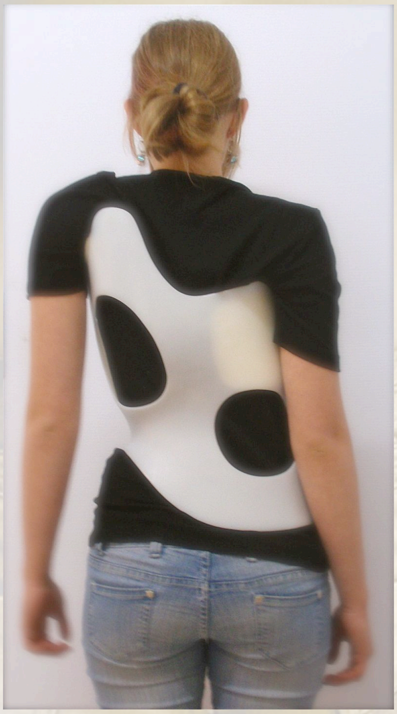

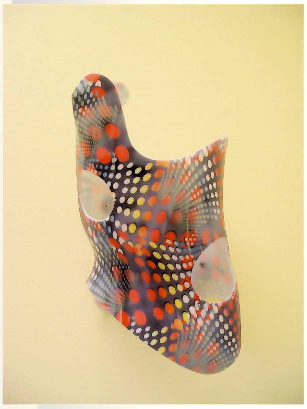

The lumbar counter curve made it necessary to counter tilt the pelvic area in order to correct the stiff thoracic curve at it's best (shift to the left).

© Hans-Rudolf Weiss, MD, 2010

4-curve double major (4C)

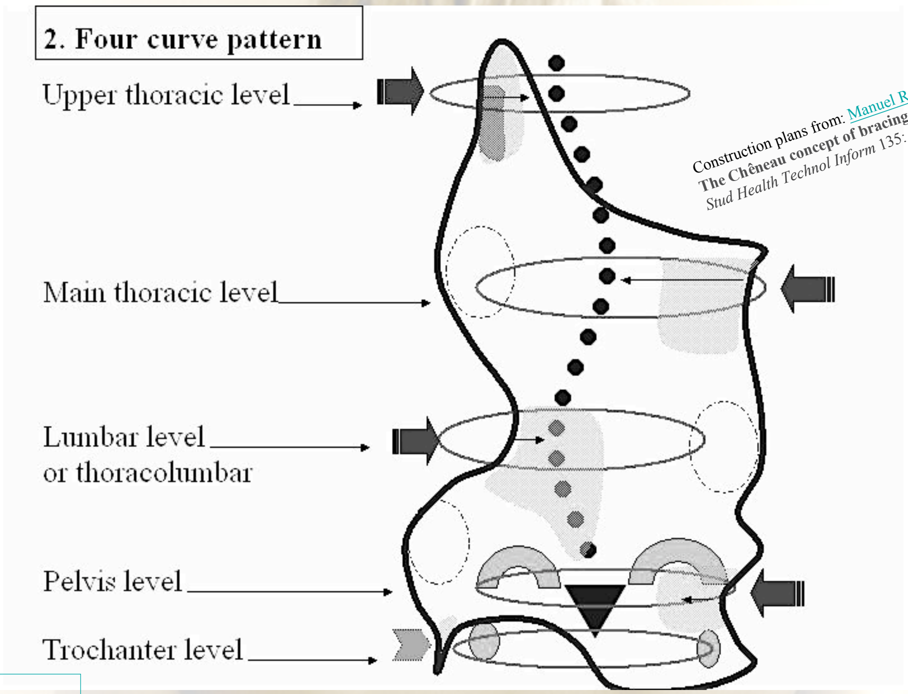

Construction plans from: Manuel Rigo, Hans-Rudolf Weiss (2008)
The Chêneau concept of bracing--biomechanical aspects.
Stud Health Technol Inform 135: 303-319

Trochanteric pressure area may be omitted in modern well balanced braces!

Example of treatment

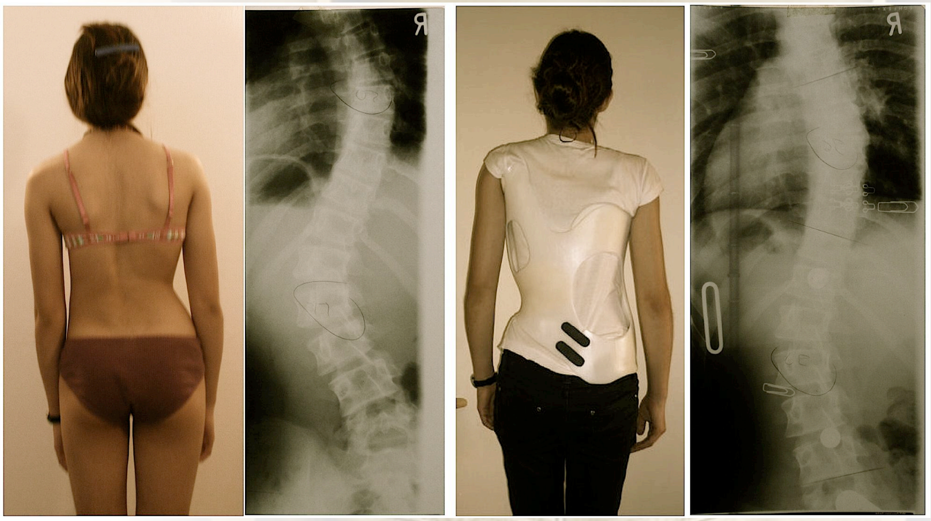

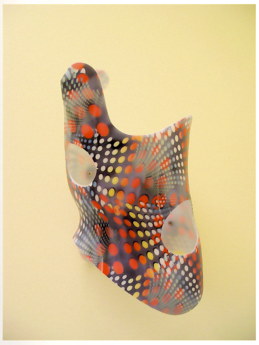

Girl from Danmark with a significant 4C (double major). The high lumbar apex in combination with a mid / low thoracic apex usually does not allow to correct very much. As the girl was 12 years at the start of treatment, a sufficient in-brace correction has been achieved.

© Hans-Rudolf Weiss, MD, 2010

4-curve single lumbar (4CL)

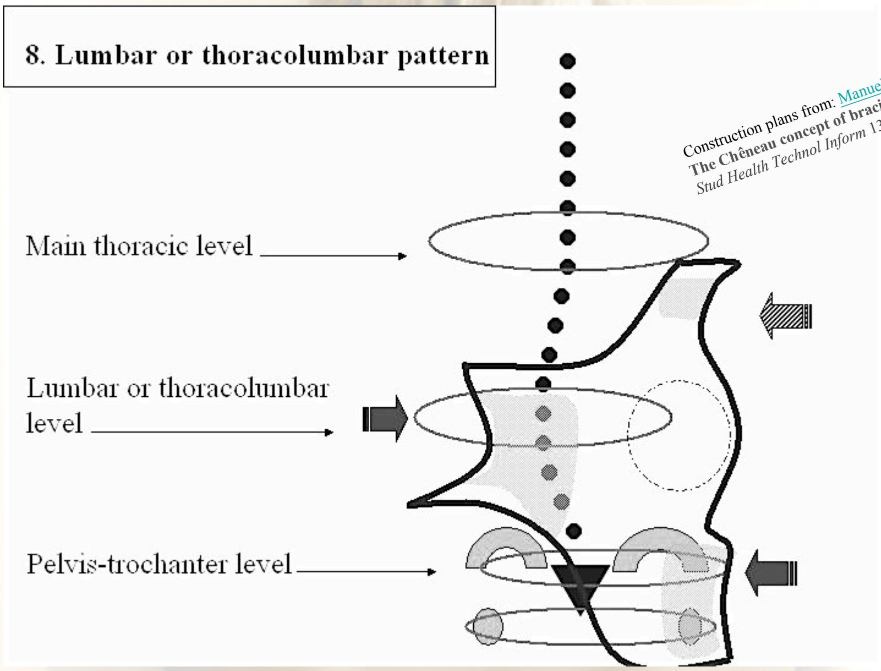

Construction plans from: Manuel Rigo, Hans-Rudolf Weiss (2008)
The Chêneau concept of bracing--biomechanical aspects.
Stud Health Technol Inform 135: 303-319

Example of treatment

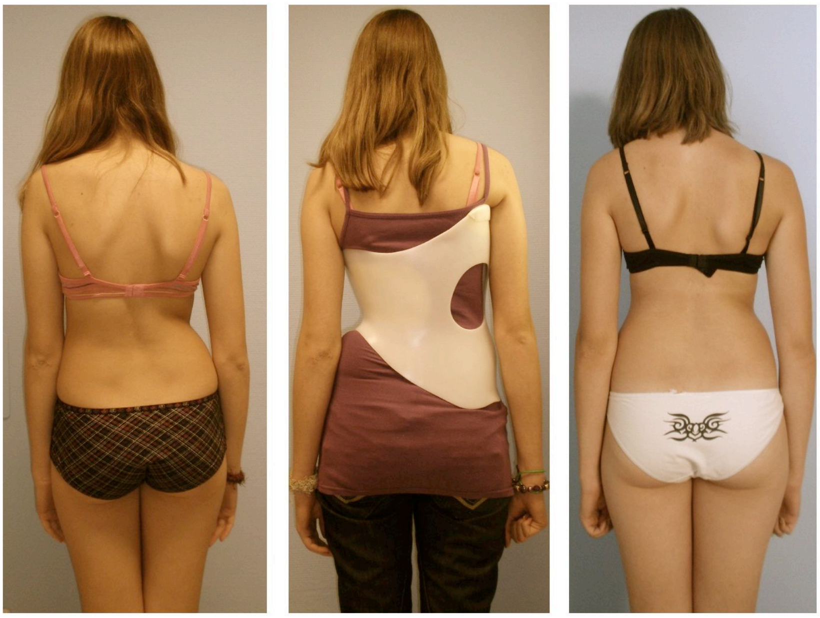

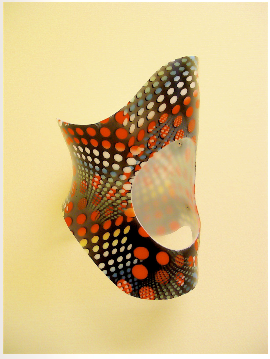

14 year old girl with a 4CTL curve clearly overcompensated allowing best possible realignment. After 6 months of treatment pelvis is already compensated.

© Hans-Rudolf Weiss, MD, 2010

3-curve thoracolumbar (3CTL)

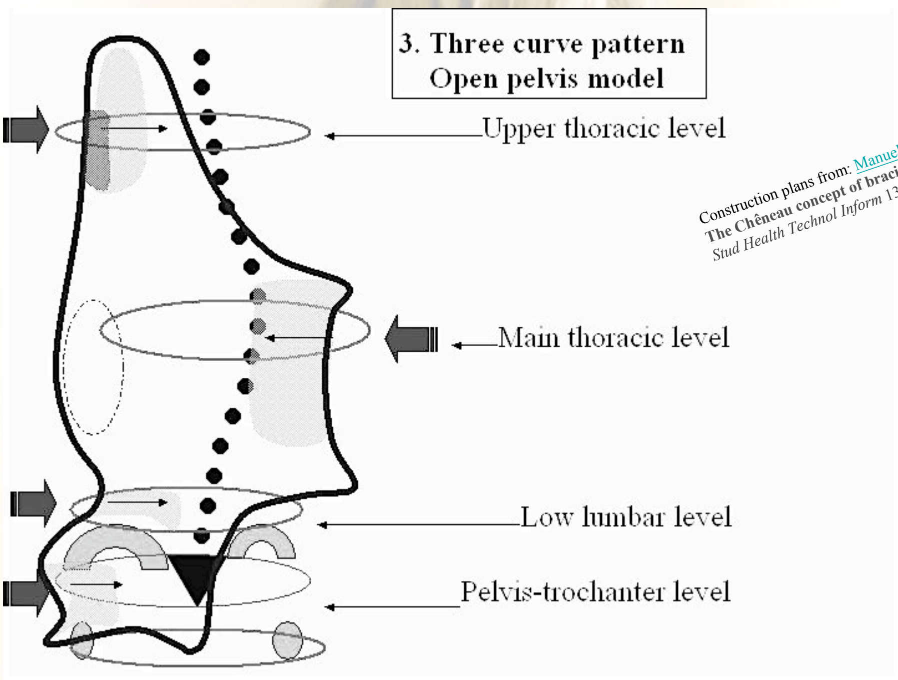

Construction plans from: Manuel Rigo, Hans-Rudolf Weiss (2008)
The Chêneau concept of bracing--biomechanical aspects.
Stud Health Technol Inform 135: 303-319

Example of treatment

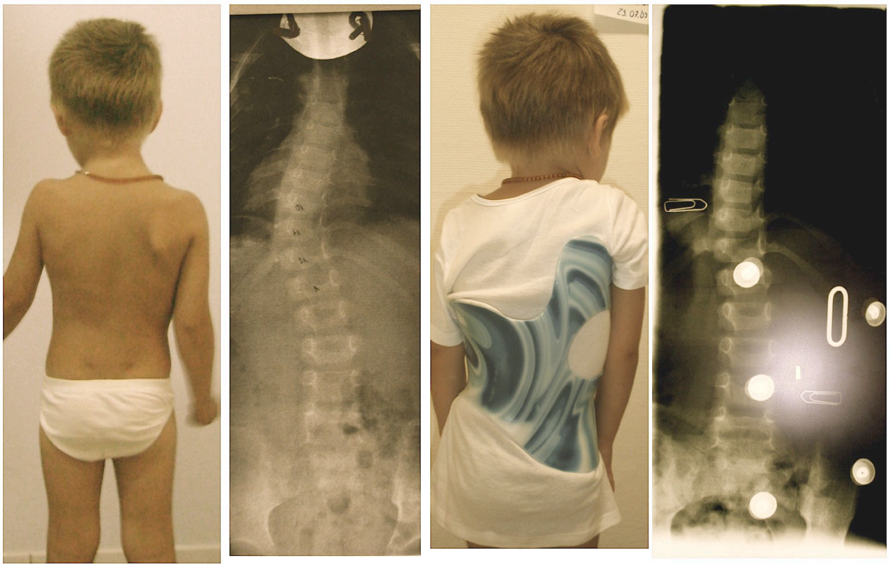

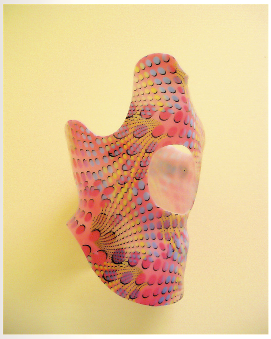

Italian boy, born 20.12.05 with 28° Cobb before the adjustment of his second brace on the left / in the Gensingen brace® on the right 6° Cobb (Initial Chêneau brace treatment started at the age of 4 years with 40°)

© Hans-Rudolf Weiss, MD, 2010
