# Supplementary material for: "Brace technology" thematic series - the Gensingen brace™ in the treatment of scoliosis
Source: Scoliosis. 2010 Oct 13;5:22. doi: 10.1186/1748-7161-5-22 (PMC2967515; doi:10.1186/1748-7161-5-22)
Supplement: Additional file 5 — Physicians Checklist as used in German. The checklist is in German language and serves only for documentation purposes within this article. [file 1748-7161-5-22-S5.PDF]

Dr. med. Hans Rudolf Weiß
Arzt für Orthopädie, Physikalische & Rehabilitative Medizin
Chirotherapie, Physikalische Therapie.
Spezialgebiet Wirbelsäulendeformitäten & Schmerzsyndrome
Alzeyer Straße 23, 55457 Gensingen

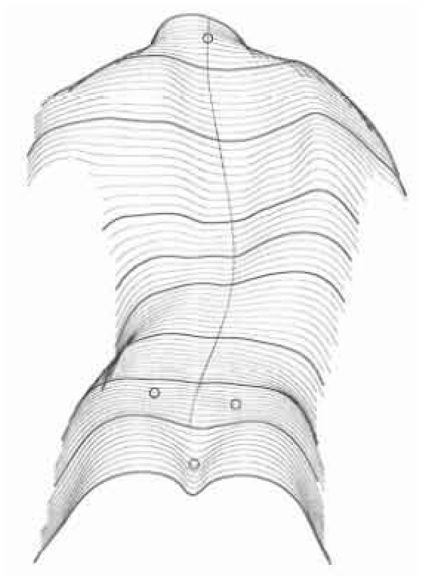

Abnahmeprotokoll für Skolioseorthesen (Stand 4/09) © Dr. Weiss 2009

Name: Vorname geb,:

Diagnose: Krümmungsmuster (Schlüsselmuster nach Rigo):

Schmerzen i. K. ☐ Atemnot i. K. ☐ Verordnungsdatum:

	Optimal	ausreichend	nicht sicher	gar nicht
Krümmungsmuster getroffen:	☐	☐	☐	☐
Spiegelung erreicht:	☐	☐	☐	☐
Sagittalprofil physiologisch	☐	☐	☐	☐
Freiräume:	☐	☐	☐	☐
Beckenhyperkompensation: *(Muster L, TL und DM)*	☐	☐	☐	☐
Freiraum CI kaudal Lumbaleinschnitt: *(Muster L, TL und DM)*	☐	☐	☐	☐
Freiraum ventral Lumbal-DZ: *(Muster L, TL, DM)*	☐	☐	☐	☐
11. Rippe frei: *(Muster T2, T6, DM1)*	☐	☐	☐	☐
Thorakalpelotte kranial augmentiert: *(Muster T2, T6, DM1, DM2)*	☐	☐	☐	☐
Freiraum Th mittlere Axillarlinie – parasternal Gegenseite: *(Muster T2, T6, DM1, DM2)*	☐	☐	☐	☐
Axillareinrichtung (kranial und zentral): *(Muster T2, T6, DM1, DM2)*	☐	☐	☐	☐
Freiraum zwischen Punkt 3 und 4(nach Chêneau): *(Muster T2, T6, DM1, DM2)*	☐	☐	☐	☐

Das Korsett konnte ohne Änderung ☐ **mit Änderungen** ☐ **nicht** ☐
abgenommen werden!

Neuversorgung erforderlich wegen Konstruktionsmängeln ☐ **wegen Wachstum** ☐

Gensingen, den
